# Gelatin-Based Hydrogels through Homobifunctional Triazolinediones Targeting Tyrosine Residues

**DOI:** 10.3390/molecules24030589

**Published:** 2019-02-07

**Authors:** Roberto Guizzardi, Luca Vaghi, Marcello Marelli, Antonino Natalello, Ivan Andreosso, Antonio Papagni, Laura Cipolla

**Affiliations:** 1 Department of Biotechnology and Biosciences, University of Milano—Bicocca, Piazza della Scienza 2, 20126 Milano-IT, Italy; r.guizzardi@campus.unimib.it (R.G.); antonino.natalello@unimib.it (A.N.); 2 Department of Materials Science, University of Milano—Bicocca, via R. Cozzi 55, 20125 Milano-IT, Italy; luca.vaghi@unimib.it (L.V.); i.andreosso@campus.unimib.it (I.A.); 3 CNR, Institute of Molecular Science and Technologies, Via C. Golgi 19, 20133 Milano-IT, Italy; m.marelli@istm.cnr.it

**Keywords:** gelatin, hydrogel, ene-type chemistry, tyrosine, triazolinediones, cyclic diazodicarboxamides, chemical cross-linking, natural polymers

## Abstract

Gelatin is a biopolymer with interesting properties that can be useful for biomaterial design for different applications such as drug delivery systems, or 3D scaffolds for tissue engineering. However, gelatin suffers from poor mechanical stability at physiological temperature, hence methods for improving its properties are highly desirable. In the present work, a new chemical cross-linking strategy based on triazolinedione ene-type chemistry towards stable hydrogel is proposed. Two different homobifunctional 1,2,4-triazoline-3,5(4*H*)-diones, namely 4,4′-hexane-1,6-diylbis(3*H*-1,2,4-triazoline-3,5(4*H*)-dione) **1** and 4,4′-[methylenebis(4,1-phenylene)]*bis*(3*H*-1,2,4-triazoline-3,5(4*H*)-dione) **2** were used as cross-linkers in different ratio to tyrosine residues in gelatin. The reaction was proved effective in all experimented conditions and hydrogels featured with different thermal stability were obtained. In general, the higher the cross-linker/tyrosine ratio, the more thermostable the hydrogel. The swelling properties are strictly dependent upon the chemical nature of the cross-linker.

## 1. Introduction

Gelatin is a protein mixture obtained from collagen hydrolysis in acid or basic conditions, with excellent properties in terms of biodegradability, biocompatibility, cell-adhesion features, and ease of modification, and it is also non-immunogenic. Due to its properties together with its inexpensiveness and readiness, chemically modified gelatin or gelatin blended with other natural or unnatural (macro)molecules [1,2,3] have been extensively employed as biomaterials for tissue engineering [4,5] and for drug delivery [6]. However, the main limitation in gelatin biomaterial design concerns its native poor mechanical properties and short degradation times, especially under physiological conditions [7,8,9], posing difficulties for shaping gelatin into hydrogels and scaffolds with stable morphologies and the desired mechanical features. In order to overcome this drawback, gelatin-based biomaterials are usually the result of physical, enzymatic, or chemical cross-linking [10,11,12,13]. In the crosslinking process, the biopolymer functional groups react chemically, enzymatically or physically interact with the cross-linker of choice, affording a 3D network. Chemical cross-linking is generally preferred, since it affords stable covalent cross-links and better tuning and reproducibility of the process. Chemical cross-linking may exploit either the intrinsic reactivity of functional groups present in the biopolymer (i.e., amino acids side chain) [14,15], or the extrinsic reactivity of functional groups introduced ad hoc in the biopolymer, that can be lately reacted bio-orthogonally [16]. The second approach often relies on the so-called click-reactions [17,18] based for example on carbonyl/oxime-hydrazone chemistry [19,20], Staudinger reaction [21,22], Huisgen-type cycloaddition [23,24,25], Diels-Alder [26,27,28], and thiol-ene addition [29,30]. Click-chemistry offers several advantages such as high yield, mild reaction conditions and chemoselectivity. Despite the very effective chemistry beyond extrinsic bioorthogonal reactions [31], and its broad applicability to several fields [32], the main limitation is due to the need of a two step process, the first of which is the introduction of extrinsic functionalities by chemical or enzymatic modification or by genetic engineering approaches.

Gelatin has been cross-linked by taking advantage of extrinsic functional groups [33,34,35,36,37,38] or through direct cross-linking based on intrinsic amino acid reactivity. The most common cross-linkers used for gelatin [12] are glutaraldehyde [39,40], 1,4-butanediol diglycidyl ether (BDDGE) [10], genipin [41], citric acid [42], and bisvinyl sulfonemethyl (BVSM) [43]. All of the above mentioned cross-linking agents target amino, hydroxyl or carboxyl groups in the amino acid side chains. Less exploited are protein cross-linking techniques targeting the aromatic ring of tyrosine residues. Among them, oxidative cross-linking of tyrosine phenolic groups has been proposed, mimicking the well-known natural oxidation process of phenolic moieties [44,45,46]. In addition to the oxidative coupling affording zero-length dityrosine adducts, very recently triazolinedione chemistry [47] has been proposed.

Triazolinedione ene-type chemistry recently emerged as a click-reaction for the bioconjugation to tyrosine residues, mediated by cyclic monofunctional diazodicarboxamides, such as 4-phenyl-1,2,4-triazoline-3,5-dione (PTAD) [48,49,50,51]. Hetherobifunctional triazolinediones (TADs), have been used for the synthesis of DNA−protein conjugates [52], while homobifunctional TADs have been applied to the cross-linking of synthetic polypetides [53].

In the present work, we propose for the first time the use of homobifunctional TADs for gelatin cross-linking towards the production of hydrogels and scaffolds.

## 2. Results and Discussion

### 2.1. Cross-Linking of Gelatin

**Figure 1:**
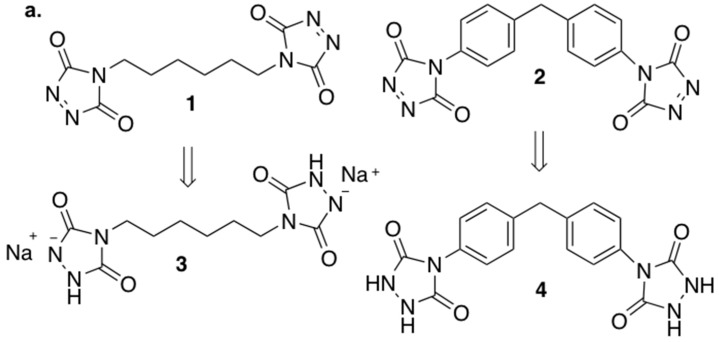

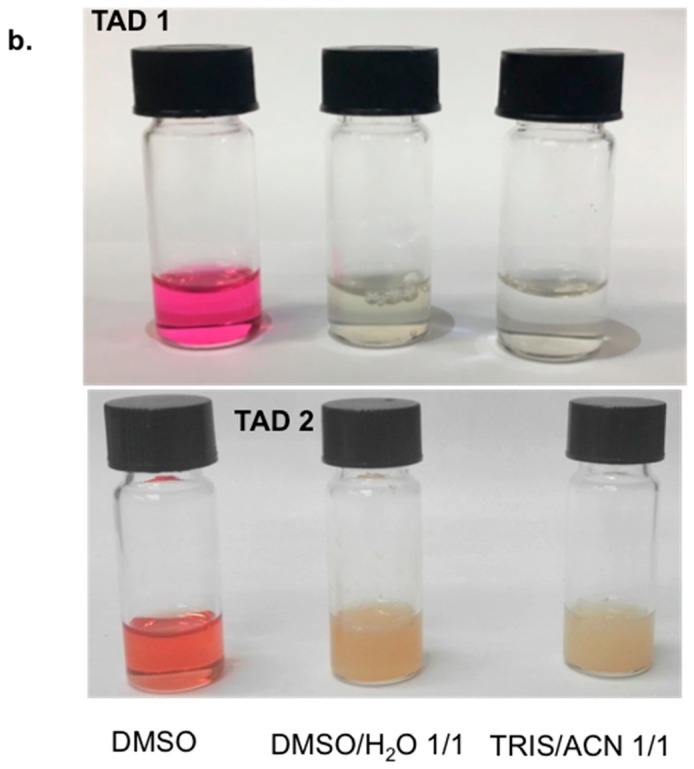
(**a**) The gelatin cross-linkers hetherobifunctional triazolinediones (TADs) **1** and **2** and the corresponding reduced forms **3** and **4,** respectively; (**b**) TADs stability in different solvents (DMSO, 1:1 DMSO/H_2_O, 1:1 TRIS Buffer solution-pH = 7.4/acetonitrile). The disappearance of the fuchsia color indicates the degradation of **1** and **2**.

Two different homobifunctional reagents were chosen as protein cross-linkers, both characterized by two terminals 1,2,4-triazoline-3,5(4*H*)-diones groups, namely 4,4′-hexane-1,6-diylbis(3*H*-1,2,4-triazoline-3,5(4*H*)-dione) **1** and 4,4′-[methylenebis(4,1-phenylene)]*bis*(3*H*-1,2,4-triazoline-3,5(4*H*)-dione) **2** (Figure 1a). Reagents **1** and **2** were synthesized following literature methodologies (see Supplementary Material for details) [54,55].

Gelatin can be easily dissolved in aqueous solutions above 37 °C; however, TADs are reported to undergo degradation in water [50], despite a certain stability of some TADs in aqueous medium reported in bioconjugation reactions by PTAD [56] or in cross-linking reaction of a synthetic Lys-Tyr polypeptide in TRIS buffer solution by difunctional TAD **2** [53]. In our hands both **1** and **2** almost immediately decomposed in any type of aqueous environment, as clearly indicated by the disappearing of their characteristic fuchsia color in a few seconds (Figure 1b). Thus, suitable reaction conditions should be found for effective gelatin cross-linking by TADs, in order to allow the cross-linking agents to react with tyrosine residues present in the protein, affording the desired chemical reaction towards network formation. Gelatin can be dissolved in DMSO, after vigorous stirring for 3 h at 37 °C at a concentration of 12 mg/mL; in addition, DMSO has proven to be fully compatible with the use of TADs [50]: DMSO solutions of **1** and **2** are stable for several hours since the characteristic fuchsia color is maintained, confirming the stability of the two cross-linking agents in this medium (Figure 1b). Thus DMSO could be the solvent of choice for the gelatin cross-linking reaction. Given the amino acid composition of porcine gelatin, tyrosine is expected to be present from 3 to 4 mmol per 100 g of dry gelatin [57]. In order to check the efficacy of the cross-linking, different TADs/tyrosine ratios were used. In a typical experiment, 100 mg of gelatin are dissolved in DMSO (8 mL) at 37 °C; after complete dissolution, the solution is cooled to r.t. and kept in the dark, due to the thermal and photochemical instability of **1** and **2** [58], and 0.5:1, 1:1, 2:1, 5:1 TADs/tyrosine molar ratio was added. The reaction mixtures slowly discolored over 30 min of stirring, indicating the progress of the reaction. Upon completion of the reaction (indicated by the disappearance of the TAD color), cross-linked gelatin was recovered by precipitation (adding methanol in the case of **1**, and acetone in the case of **2**).

**Figure 2:**
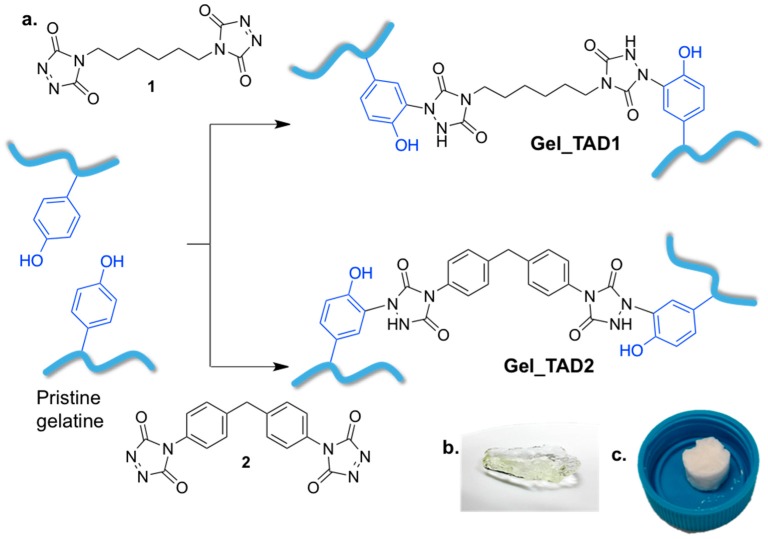
(**a**) cross-linking reaction between gelatin and **1** or **2**; (**b**) recovered hydrogels; (**c**) dried cross-linked gelatin hydrogels.

Cross-linked gelatin hydrogels (Figure 2) were characterized by their thermal resistance at pH 7.4 and 37 °C, swelling properties, FT-IR spectroscopy, and SEM. All the collected data demonstrated the effectiveness of the cross-linking methodology. Cross-linked gelatin shows improved thermal stability as TADs/tyrosine ratio increases, as a consequence of increased reticulation.

In order to demonstrate that cross-linking occurs through covalent bonds formation (chemical cross-linking), instead of non-bonding interactions (i.e., hydrogen bonds, physical cross-linking), gelatin was also treated with the reduced form (urazole) of the TADs (compounds **3** and **4**, Figure 1a), in a 5 fold excess in respect to tyrosine for 30 min (as for the treatment with **1** and **2**), and identically worked up. Given that effective cross-linking renders gelatin insoluble in water at 37 °C, water solubility assay is an immediate and easy way to check the cross-linking. Thus gelatin treated with **3** and **4** was soaked in water at 37 °C; the specimen promptly dissolved in water at 37 °C, indicating that physical cross-linking did not occur. This assay demonstrates that chemical covalent cross-linking is actually occurring with TADs **1** and **2**.

### 2.2. Characterization of Cross-Linked Gelatin

#### 2.2.1. Thermal Stability

**Figure 3:**
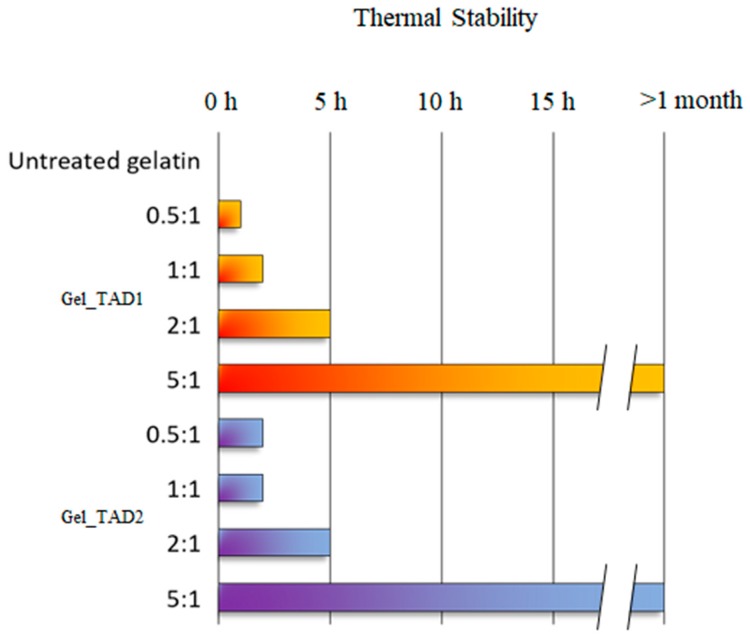
Thermal stability of hydrogels at 37 °C; results for any TAD/tyrosine ratio are means of 3 independent experiments.

Freeze-dried cross-linked gelatin specimens were rehydrated with 1 mL of PBS buffer (pH = 7.4), placed in a 37 °C chamber to test their thermal stability (Figure 3), and compared with pristine gelatin samples. As expected, pristine gelatin dissolved almost immediately; cross-linked gelatin with TADs/tyrosine 0.5:1 ratio displayed better resistance when compared to untreated gelatin, dissolving in about 1 h when reacted with TAD **1** and 2 h with TAD **2,** respectively. In general, it was observed that the increase of TADs/tyrosine ratios affords better performing cross-linked gelatin. For TAD/tyrosine ratio 5:1, cross-linked gelatin was stable over a month.

#### 2.2.2. Swelling Properties

**Figure 4:**
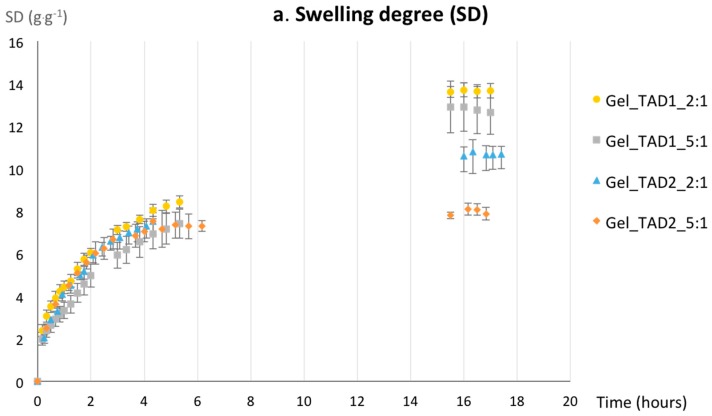

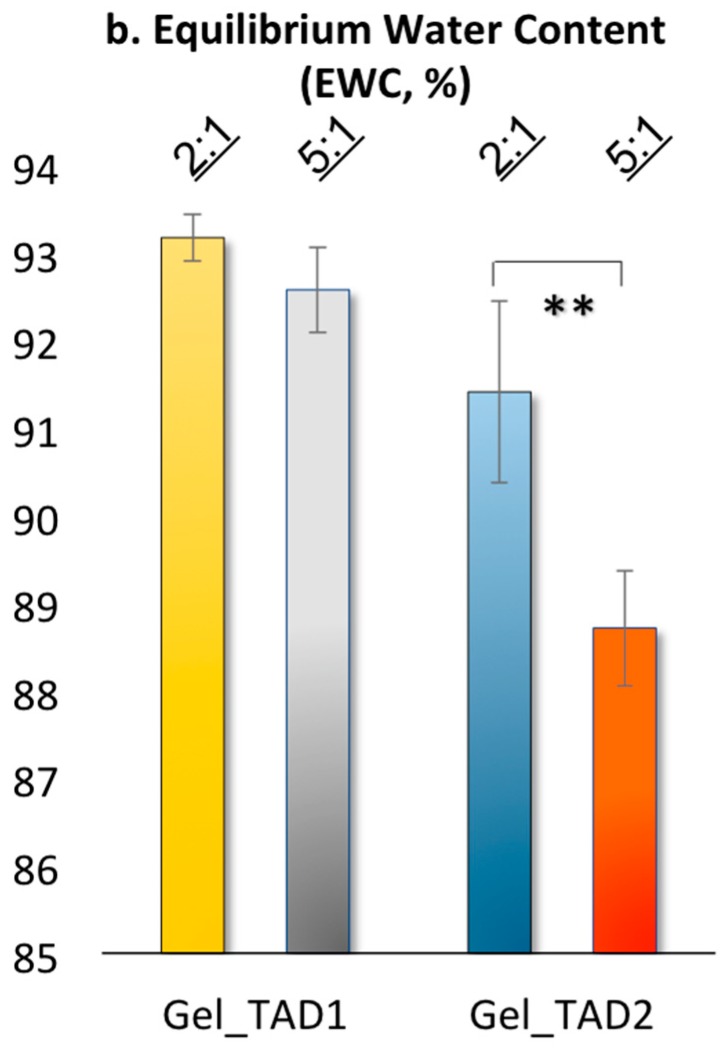
(**a**) Swelling degree for gelatin hydrogel cross-linked either with TAD **1** or **2** at TAD/tyrosine molar ratio 2:1 and 5:1; (**b**) equilibrium water content; data are average of three independent experiments, bars indicate standard deviation and statistical analysis was performed with t-student with ** *p* < 0.01).

Based on thermal stabilities, cross-linked gelatin with TADs/tyrosine 2:1 and 5:1, which resulted the more stable, were tested for their swelling behavior in water by gravimetric analysis. Gel swelling properties are usually dependent upon several factors, including pore size of the network, interactions between the network (polymer chains and cross-linkers) and the solvent, and chain mobility during the swelling process [59]. The dynamic swelling properties (swelling degree, SD) and the equilibrium water content (EWC) for **Gel_TAD1** and **Gel_TAD2** are reported in Figure 4. All of the hydrogel samples were prepared of same dimensions, approximately (10 mm diameter × 5 mm height), as described in the Experimental Section.

The SD plot (Figure 4a) shows a similar kinetic behavior for all of the considered specimen within the first 5 h. However, **Gel_TAD1** samples have better water retaining properties if compared to **Gel_TAD2**, requiring higher times for reaching the equilibrium. This behavior might be ascribed to the different hydrophilicity of the linkers and eventually to the different conformational freedom that may have a role in the chain mobility of the network. The diphenyl moiety in TAD **2** is more hydrophobic and confers higher conformational rigidity to the cross-linker if compared to the hexyl moiety in TAD **1**. In addition, the chemical nature of TAD **2** has more relevance in influencing gel properties as a function of TAD/Tyr ratio: the higher the TAD/Tyr ratio, the lower SD and EWC values.

#### 2.2.3. FT-IR Characterization

**Figure 5:**
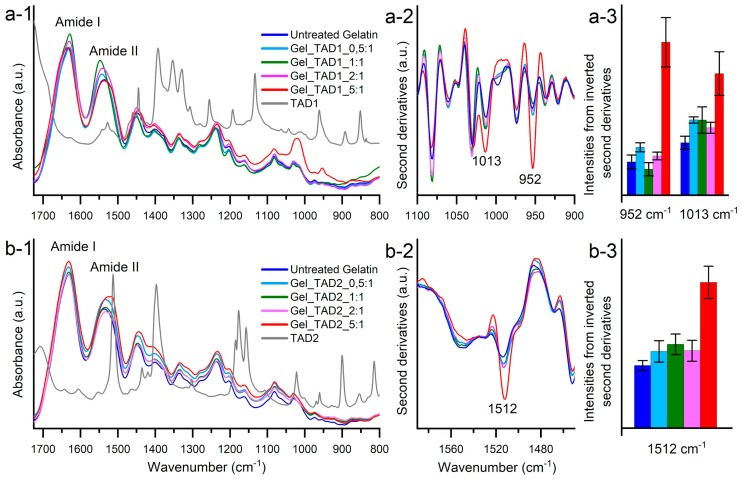
Insets **a-1** and **b-1**: ATR-FTIR absorption spectra of untreated gelatin, TADs and cross-linked gelatin specimens reported in the 1725–800 cm^−1^ region.; the second derivatives of TAD **1** (inset **a-2**) and TAD **2** (inset **b-2**) cross-linked samples are reported in the spectral regions where the contributions of the TADs moieties can be detected. The intensities of the indicated components were evaluated from the second derivative spectra (insets **a-3** and **b-3**). Error bars refer to three independent measurements. Spectra are shown after normalization at the Amide I band area.

Gelatin specimens were analyzed by FTIR measurements in attenuated total reflection (ATR). The ATR-FTIR absorption spectra of the different gelatin samples display the typical spectral features of polypeptides and are characterized by the Amide I and Amide II bands and several partially overlapped components in the fingerprint region around 1500–800 cm^−1^ (Figure 5, insets a-1 and b-1). Small spectral changes were observed after TAD **1** and TAD **2** treatments. The intensity variations were evaluated by second derivative analysis that enable to discriminate among overlapped components of the absorption spectra. In particular, a significant increase of the 1013 cm^−1^ and 952 cm^−1^ peaks was observed in the 5:1 TAD **1**/tyrosine ratio (Figure 5, insets a-2 and a-3). These components, which are also present in the neat TAD **1** cross-linker (Figure 5, inset a-1), were tentatively assigned to the CN vibrations [60,61]. In the case of TAD **2** cross-linker, a strong increase of the 1512 cm^−1^ component was observed in the 5:1 TAD **2**/tyrosine ratio (Figure 5, insets b-2 and b-3). This component is typically assigned to the CC vibrations of the aromatic ring [61,62]. The FTIR data thus confirm the gelatin cross-linking through TAD **1** and TAD **2**.

#### 2.2.4. Scanning Electron Microscopy Micrographs

**Figure 6:**
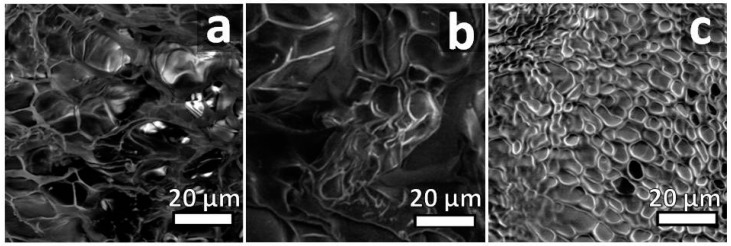
Representative SEM micrograph of gelatin samples (**a**) pristine gelatin and cross-linked with **1** in (**b**) 0.5:1 and (**c**) 5:1 TAD **1**/tyrosine ratio respectively.

Low-vacuum scanning electron microscopy was used in order to investigate the surface morphological changes induced by the different TADs cross-linkers and ratio experimented. A porous structure along with the foam-like morphology was recognizable (Figure 6 and Supplementary Material, Figure S5). As a general trend, moving from 0.5:1 to 5:1 TAD/tyrosine ratio (Figure 6, and Supplementary Material, Figure S5) the increased cross-linkers amount results in an increase in the reticulation texture and a less heterogeneity in porosity and pore size due to increased cross-linking, while pristine gelatin shows a heterogeneous texture with open pores ranging from 5 to 20 µm (Figure 6a). The addition of the cross-linkers increases the interconnected porosity and the pore size shrinks down, ranging from 5 to 2 µm.

## 3. Materials and Methods

### 3.1. General

All reagents and solvents were purchased from commercial sources (Sigma-Aldrich S.r.l., Milan, Italy and Fluorochem Ltd., Hadfield, United Kingdom) and used without further purification. Gelatin from porcine skin-Type A was used for hydrogel preparation (Sigma-Aldrich, catalog no. G2500). Tyrosine content in gelatin is reported as 3.4 μmol every 100 mg of gelatin [57]. ^1^H NMR spectra were recorded with a Bruker Avance 500 (Bruker corp., Billerica, MA, USA). ATR-FTIR spectra of TAD **1** and **2** and related synthetic intermediates were recorded with a Perkin-Elmer Spectrum 100 (Perkin-Elmer Waltham, MA, USA); ATR-FTIR spectra of gelatin specimens were collected with a Varian 670-IR (Varian Australia Pty Ltd., Mulgrave VIC, Australia) spectrometer equipped with the Quest (Specac) ATR device [63]; Scanning electron microscopy (SEM) analysis were performed with a Philips XL30 ESEM (FEI, Hillsboro, OR, USA).

Cross-linked gelatin was freeze-dried by a Christ alpha 1–2 freeze dryer (Christ, Osterode am Harz, Germany). Melting points were measured with a Stanford Research Systems Optimelt apparatus.

### 3.2. Synthesis of Cross-Linking Agents

Cross-linkers **1** and **2** were synthesized adapting literature procedures reported by Culbertson and McGrath [54]. The procedure by Mallakpour and co-worker was used for the oxidation of urazoles **3** and **4** [55]. For sake of completeness synthetic procedures are reported in the Supplementary Material.

### 3.3. Gelatin Cross-Linking

#### 3.3.1. Preparation of TAD 1 Cross-Linked Hydrogel Gel_TAD1

To 100 mg of gelatin, 8 mL of DMSO were added and the suspension heated to 37 °C until complete dissolution. Given tyrosine content, for the 0.5:1 and 1:1 TAD/Tyr hydrogel, 95 μL and 191 μL of a freshly prepared 5 mg/mL TAD **1** solution in DMSO were added respectively (corresponding to 1.7 and 3.4 μmol of TAD **1**); for the 2:1 and 5:1 molar ratio, 127 μL and 318 μL of a freshly prepared 15 mg/mL TAD **1** solution in DMSO were added respectively (corresponding to 6.8 and 17.0 μmol of TAD **1**). The solutions were reacted at r.t. in the darkness under stirring until the purple solution turned colorless (30 min). Cross-linked gelatin was recovered by precipitation by the addition of 8 mL of methanol. The suspension was centrifuged (6500 rpm, 45 min) and washed first with methanol (5 mL) then with deionized water (2 mL × 2). The hydrogels formed on the bottom of the centrifuge tubes were freeze-dried and used for further characterization.

#### 3.3.2. Preparation of TAD 2 Cross-Linked Hydrogel Gel_TAD2

As described for Gel_TAD1, for the 0.5:1 and 1:1 TAD/Tyr hydrogel, 63 μL and 126 μL of a freshly prepared 10 mg/mL TAD **2** solution in DMSO were added respectively (corresponding to 1.7 and 3.4 μmol of TAD **2**) to the gelatin solution; for the 2:1 and 5:1 molar ratio, 126 μL and 315 μL of a freshly prepared 20 mg/mL TAD **2** solution in DMSO were added respectively (corresponding to 6.8 and 17.0 μmol of TAD **2**). The solutions were reacted at r.t. in the darkness under stirring until the purple solution turned colorless (30 min). Cross-linked gelatin was recovered by precipitation by the addition of 8 mL of acetone. The suspension was centrifuged (6500 rpm, 45 min) and washed first with acetone (5 mL) then with deionized water (2 mL × 2). The hydrogels formed on the bottom of the centrifuge tubes were freeze-dried and used for further characterization.

### 3.4. Thermal Stability Studies

Three replicas of dried Gel_TAD specimens (ca 90 mg, 10 mm diameter × 5 mm height) from each tested condition (TAD and Tyr/TAD ratio) were placed in tagged wells of 12 multiwell plates, hydrated with PBS (4 mL, pH = 7.4, physiological conditions), and kept sealed at 37 °C. The specimens were periodically visually inspected.

### 3.5. Swelling Studies

Dynamic swelling measurements were made by gravimetric measurements. Three replicas of dried 2:1 and 5:1 Gel_TAD specimens (ca 90 mg, 10 mm diameter × 5 mm height) were soaked in distilled water at 25 °C. The swollen gel discs were periodically removed from water, blotted with filter paper, and weighed on an analytical balance (Analytical Balance 220 g × 0.1 mg, Radwag AS 220/C/2) and returned to the swelling medium till the equilibrium is reached.

Swelling degree (SD) was calculated from the following equation and reported as a function of time:

Swelling degree (SD, g·g^−1^) = (W_t_ − W_0_) × W_0_^−1^. (1)

where W_t_ is the weight of swelling hydrogel at different time and W_0_ is the dry weight of the gel.

The equilibrium water content (EWC), was calculated from the following equation:

EWC (%) = (W_e_ − W_0_) × W_e_^−1^ × 100. (2)

where W_e_ is the swelling weight of the sample at equilibrium and W_0_ is the dry weight of the gel.

### 3.6. SEM Analysis

Scanning electron microscopy (SEM) analysis were performed working at 8 kV accelerating voltage and in low vacuum mode (1 Torr). Sample were dried, cut, fixed with conductive carbon tape to standard SEM stubs and directly analyzed. Working at low vacuum condition, no conductive coatings were applied in order to preserve the original structure. Samples showed good stability under electron beam illumination at the operating conditions.

## 4. Conclusions

A new cross-linking methodology for proteins such as gelatin useful for the preparation of hydrogels has been proposed through the use of homobifunctional triazolinediones. The reaction is effective, as demonstrated by several characterization techniques and hydrogel thermostability if compared to untreated gelatin.

## Supplementary Materials

The following are available online, Scheme S1: Synthesis of the cross-linking agents and experimental procedures, Figure S1: ^1^H NMR of urazole **3**, Figure S2: ^1^H NMR of urazole **4**, Figure S3: ATR-FTIR of urazole **3**, Figure S4: ATR-FTIR of urazole **4**, Figure S5: SEM images of hydrogels.
