# Supplementary material for: Gelatin-Based Hydrogels through Homobifunctional Triazolinediones Targeting Tyrosine Residues
Source: Molecules. 2019 Feb 7;24(3):589. doi: 10.3390/molecules24030589 (PMC6385110; doi:10.3390/molecules24030589)
Supplement: Supplementary file 1 [file molecules-24-00589-s001.pdf]

**Supplementary Material**

# Gelatin-based hydrogels through homobifunctional triazolinediones targeting tyrosine residues

**Roberto Guizzardi,**[1,§] **Luca Vaghi,**[2,§] **Marcello Marelli,**[3] **Antonino Natalello,**[1] **Ivan Andreosso,**[2] **Antonio Papagni,**[2,*] **Laura Cipolla**[1,*]

[1] Dept. of Biotechnology and Biosciences, University of Milano - Bicocca, Piazza della Scienza 2, 20126 Milano-IT; laura.cipolla@unimib.it
[2] Dept. of Materials Science, University of Milano - Bicocca,, via R. Cozzi 55, 20125 Milano-IT; luca.vaghi@unimib.it.
[3] CNR, Institute of Molecular Science and Technologies, Via C. Golgi 19, 20133 Milano-IT; m.marelli@istm.cnr.it.
§ These authors contributed equally to this work
* Correspondence laura.cipolla@unimib.it; Tel.: +39-0264483460

**Table of Contents**

**Scheme S1**. Synthesis of the cross-linking agents and experimental procedures.
**Figure S1**. 1H NMR of urazole **3**.
**Figure S2**. 1H NMR of urazole **4**.
**Figure S3**. ATR-FTIR of urazole **3**.
**Figure S4**. ATR-FTIR of urazole **4**.
**Figure S5**. SEM images of hydrogels.
**References**

**Synthesis of cross-linking agents**

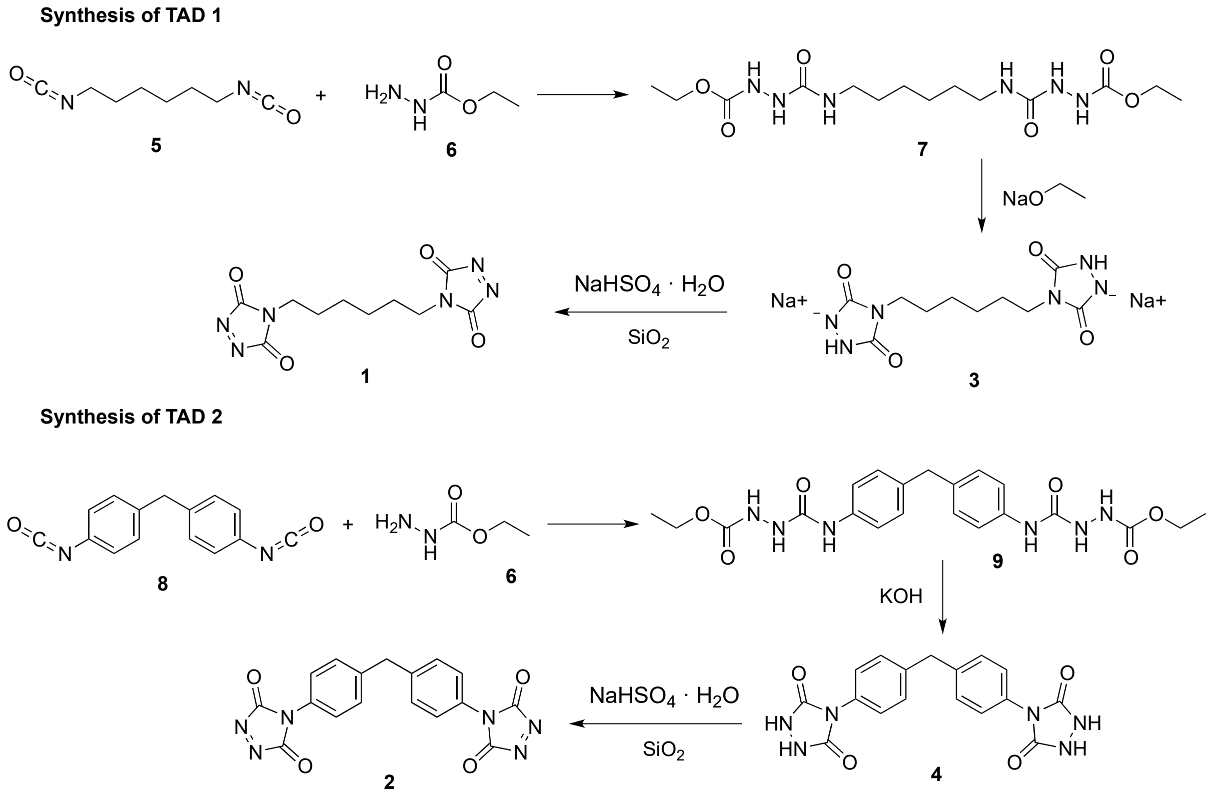


**Scheme S1**. Synthetic scheme for the synthesis of TAD **1** and **2**.

The methodologies for the synthesis of TAD **1** and **2** were adapted from the procedures reported by Culbertson and McGrath [1]. The procedure by Mallakpour and co-worker was used for the oxidation of urazoles **3** and **4** [2].

*Diethyl 4,13-dioxo-2,3,5,12,14,15-hexaazahexadecane-1,16-dioate* (**7**). Ethylcarbazate **6** (2.08 g, 20 mmol) was dissolved in 100 mL of dry toluene, under nitrogen atmosphere. The solution was cooled to 0°C, then 1,6-diisocyanate hexane **5** (1.68g, 10 mmol), in 10 ml of dry toluene, was added over a period of 10 min. The mixture was rinsed to r.t. and stirred for 30 min, then refluxed for 3 h. After cooling to r.t., the product was recovered by filtration, washed with cold toluene, and dried at 80°C overnight (3.5 g, 94%). The physical and spectroscopic data corresponded to those reported in the literature [1].

*Diethyl 2,2′-[methylenebis(4,1-phenyleneiminocarbonyl)]dihydrazinecarboxylate* (**9**). Ethylcarbazate **6** (2.08 g, 20 mmol) was dissolved in 100 mL of dry toluene, under nitrogen atmosphere. The solution was cooled to 0°C, then 1,1′- methylenebis(4-isocyanatobenzene) **8** (2.5g, 10 mmol), in 10 ml of dry toluene, was added over a period of 10 min. The mixture was rinsed to r.t. and stirred for 30 min, then refluxed for 3 h. After cooling to r.t., the product was recovered by filtration, washed with cold toluene, and dried at 80°C overnight (4.4 g, 96%). The physical and spectroscopic data corresponded to those reported in the literature [1].

*1,6-Hexanebisurazole di-sodium salt* (**3**). **7** (1.89 g, 5 mmol) and NaOEt (0.85 g, 12.5 mmol) were added to EtOH (20 mL), under nitrogen atmosphere. The reaction mixture was then heated for 24h. After 1h a clear orange solution was formed and after few hours a yellowish solid start to precipitate. The residue was collected by filtration and dried overnight at 80°C under vacuum (10 torr) affording **3** (1.6 g, 98%).The physical and spectroscopic data corresponded to those reported in the literature [1].

*4,4′-[methylenebis(4,1-phenylene)]bis(1,2,4-triazolidine-3,5-dione)* (**4**). **9** (4 g, 8.7 mmol) in KOHaq (25 mL, 4 M) was heated at 100 °C for 5 h. The mixture was then cooled with an ice bath and acidified with concentrated HCl (37%) until pH ≈ 1. The residue was collected by filtration and dried overnight at 80°C under vacuum (10 torr) (2.75 g, 86%). The physical and spectroscopic data corresponded to those reported in the literature [1].

*4,4′-hexane-1,6-diylbis(3H-1,2,4-triazole-3,5(4H)-dione)* (**1**) and *4,4′-[methylenebis(4,1-phenylene)]bis(3H-1,2,4-triazole-3,5(4H)-dione)* (**2**). Urazole (1 mmol) was grinded into a mortar with $NaHSO_4 \cdot H_2O$ (8 equiv) and $SiO_2$ (400 mg, 70 - 200 μm). Water (200 μl) was added to the fine powder obtained, and the resulting mixture was stirred for 5 min. $CH_2Cl_2$ (25 mL) was then added and the suspension was cooled to 0 °C and kept in the dark for the whole reaction time. Every 30 min $NaNO_2$ (2 equiv) was added to the reaction mixture (8 eq. overall). The inorganic salts were removed by filtration and the solvent was removed under reduced pressure affording **1** and **2** in a pure form (210 mg, 75% for **1**; 217 mg, 60% for **2**). The physical and spectroscopic data corresponded to those reported in the literature [2].

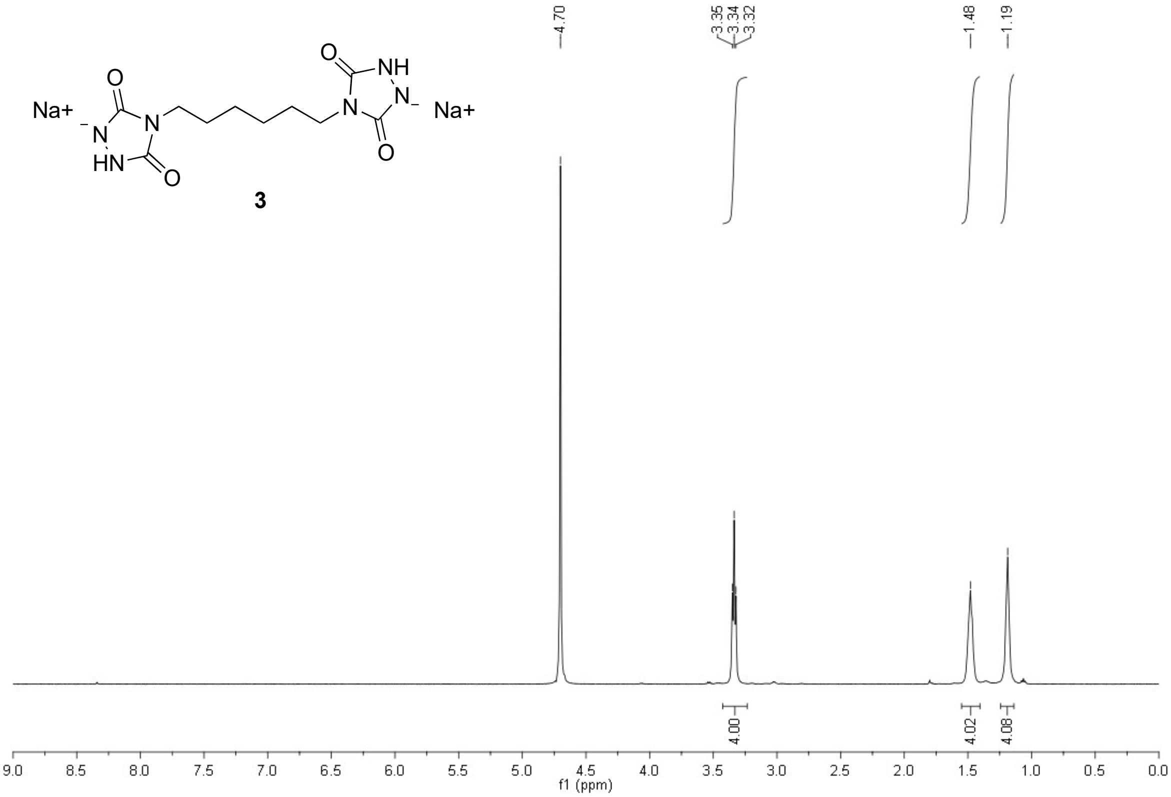


**Figure S1**. 1H NMR of urazole **3**.

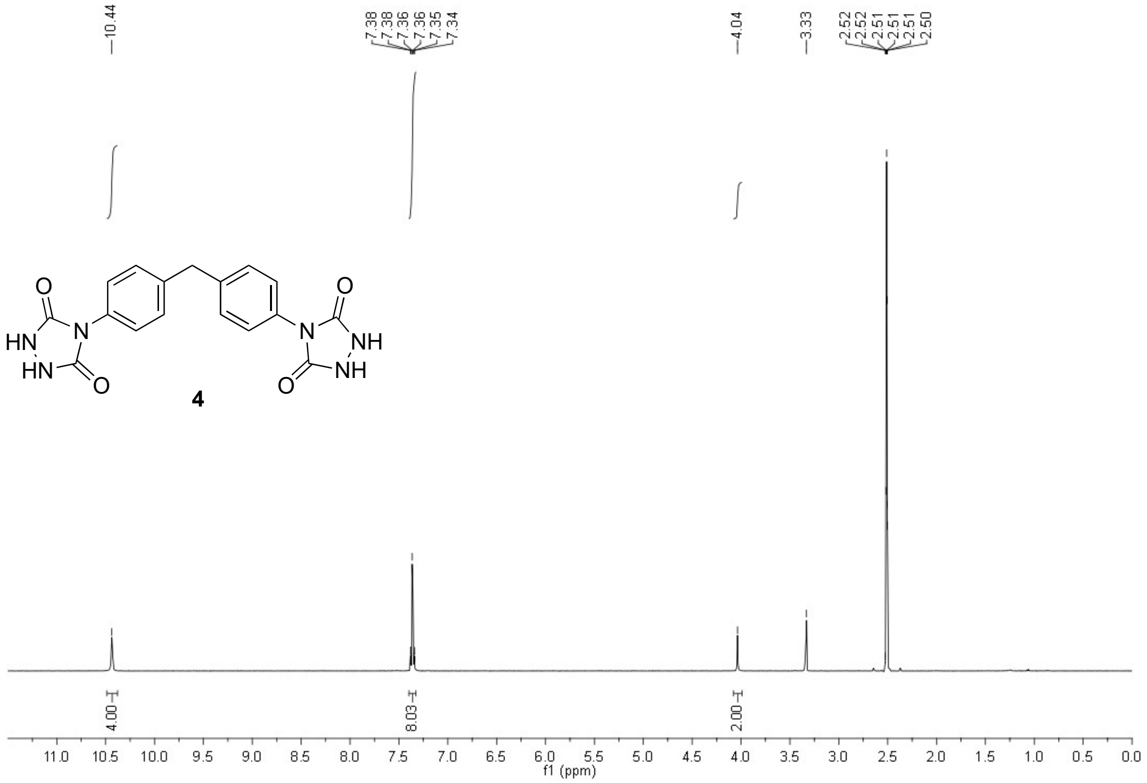


**Figure S2**. 1H NMR of urazole **4**.

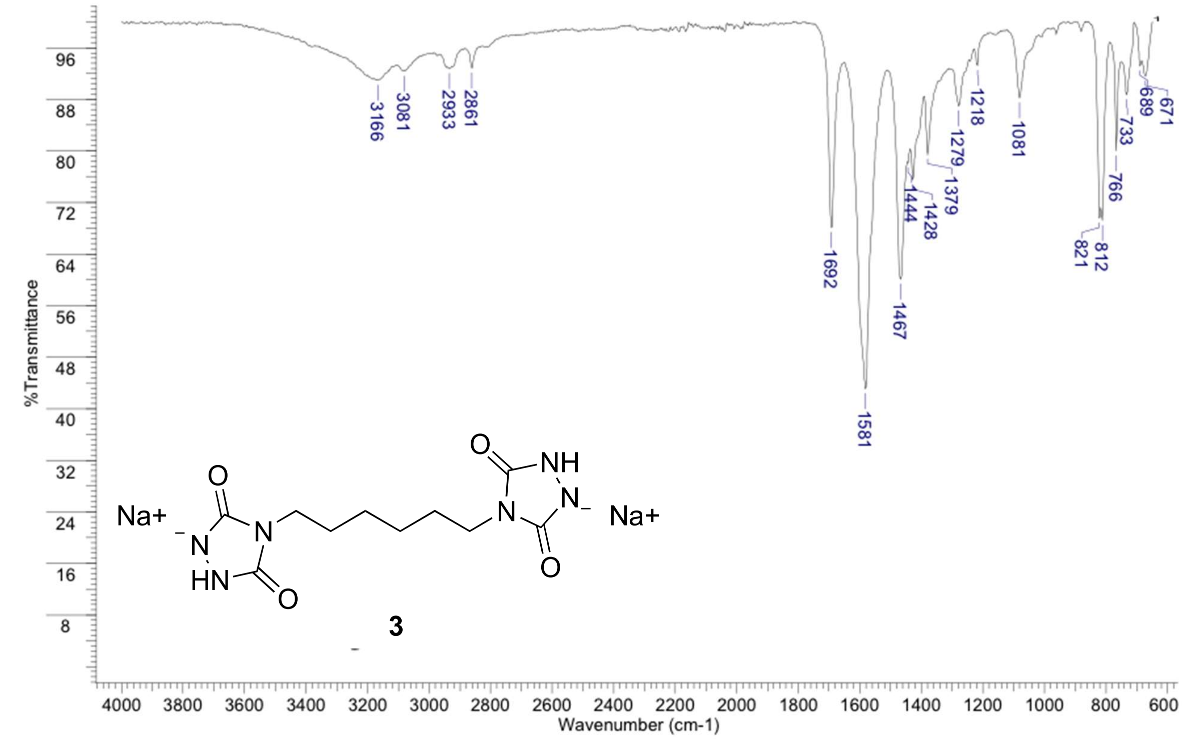


**Figure S3**. ATR-FTIR of urazole **3**.

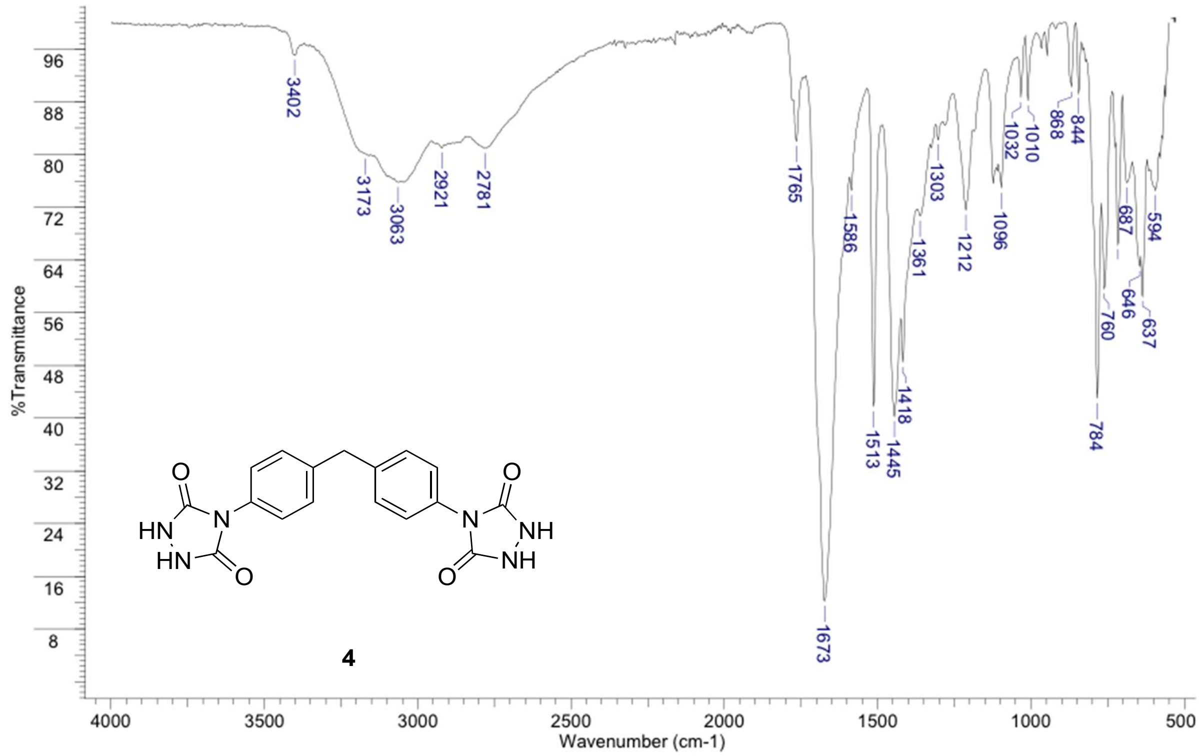


**Figure S4**. ATR-FTIR of urazole **4**.

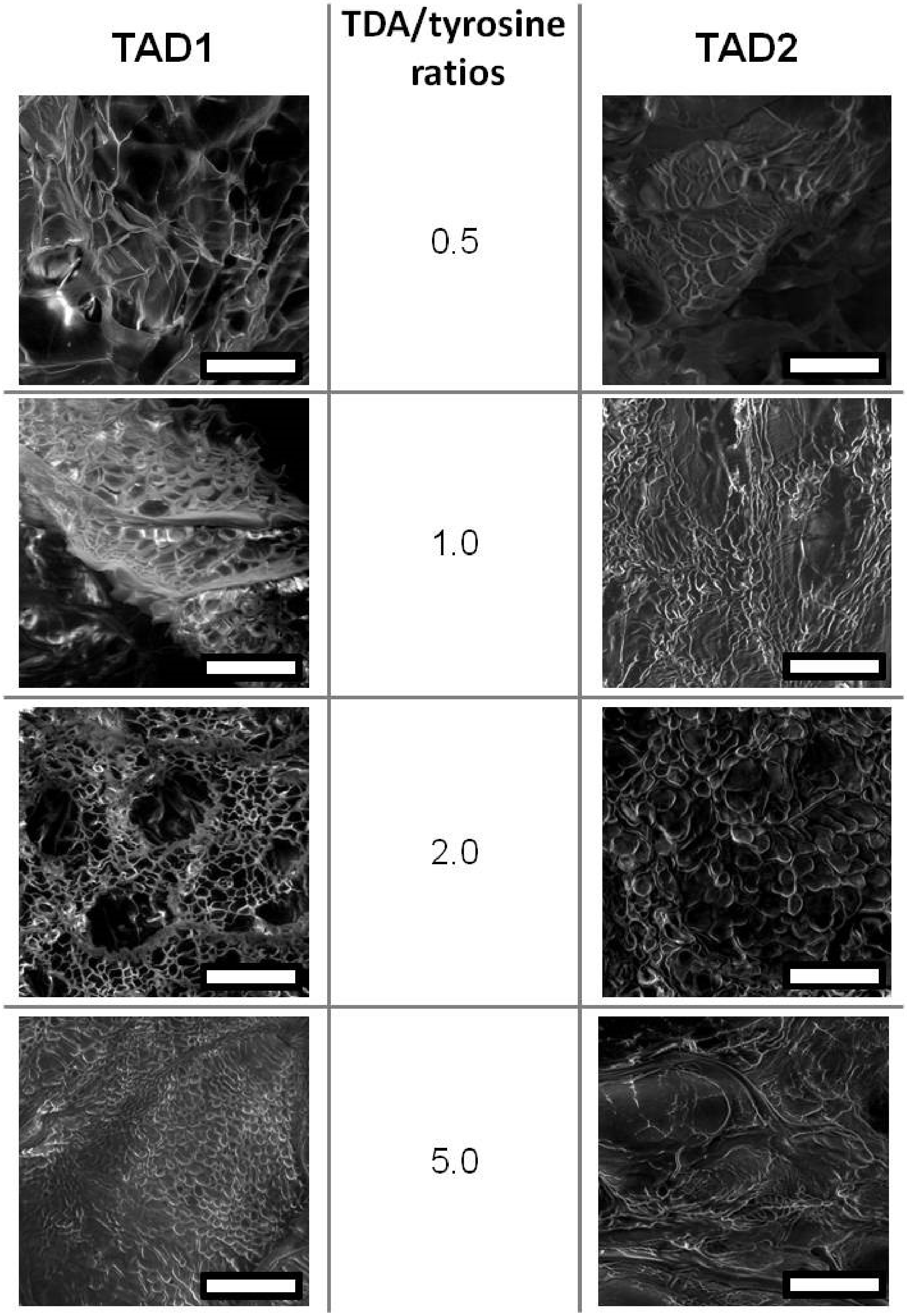


**Figure S5**. Representative SEM images for sample Gel_TAD1 e Gel_TAD2 at different TAD/tyrosine ratios. All the images were collected at the same magnification and scale bar is 50 nm.

**References**

[1] Culbertson, B.M.; McGrath, J. E.; Polymer science and technology; Plenum Press New York; 1985; Volume 31, pp. 8-11, ISBN:13:978-1-4612-9254-8.
[2] Zolfigol, M. A.; Mallakpour, S. E.; Madrakian, E.; Ghaemi, E.; Oxidation of urazoles to their corresponding triazolinediones under mild and heterogeneous conditions. *Indian J. Chem., Sect. B: Org. Chem. Incl. Med. Chem.* **2000**, *39*, 308-310, 2000:780195.
